# Do the expressions of HLA-G and killer cell immunoglobulin-like receptors change in colorectal cancer?

**DOI:** 10.55730/1300-0144.6073

**Published:** 2025-08-19

**Authors:** Ezgi DİNÇER, Fatma KAYA DAĞISTANLI, Kıvanç Derya PEKER, Damlanur SAKIZ, Figen ABATAY SEL, Demet KIVANÇ, Hayriye ŞENTÜRK ÇİFTÇİ, Çiğdem KEKİK ÇINAR, Şule KARATAŞ, Fatma SAVRAN OĞUZ

**Affiliations:** 1 Institute of Graduate Studies in Health Science, İstanbul University, İstanbul, Turkiye; 2 Department of Medical Biology, Cerrahpasa Faculty of Medicine, İstanbul University-Cerrahpasa, İstanbul, Turkiye; 3 Divison of Gastroenterology, Department of Surgery, Hisar Hospital Intercontinental, İstanbul, Turkiye; 4 Department of Pathology, University of Health Sciences Bakırkoy Dr. Sadi Konuk Training and Research Hospital, İstanbul, Turkiye; 5 Department of Medical Biology, İstanbul Faculty of Medicine, İstanbul University, İstanbul, Turkiye

**Keywords:** Human leukocyte antigen-G, killer cell immunoglobulin-like receptor, colorectal cancer

## Abstract

**Background/aim:**

The immune system functions as a well-coordinated defense mechanism, protecting the host from both external pathogens and internal threats. Cancer cells often display surface antigens that the immune system can recognize as foreign, potentially triggering an immune response. However, many cancer cells evade detection by downregulating or completely losing these surface antigens. The immune system relies on the expression of surface antigens and human leukocyte antigens (HLA) to identify and target tumor cells. One key method by which tumor cells evade natural killer (NK) cells involves alterations in HLA antigens.

Colorectal cancer (CRC) is known to cause various changes in the immune system, including the increased expression of HLA antigens on cell surfaces, reduced functionality of NK cells, and mechanisms for immune evasion.

The aim of this study was to investigate the possible roles of innate immunity and associated HLA-G molecules in the development of CRC by examining tumor tissue samples.

**Materials and methods:**

We evaluated soluble HLA-G (sHLA-G) levels via ELISA, investigated HLA-G expression loss in tumor samples through immunohistochemistry (IHC), and assessed killer cell immunoglobulin-like receptor (KIR) expression on NK cells in tumor tissues.

**Results:**

No significant correlation was found between HLA-G and sHLA-G levels (p = 0.641). Among patient samples, 16.7% (6 of 36) were positive for HLA-G, with varying intensities, while no staining was observed in control samples. Compared to control samples, IHC staining revealed a significantly higher rate of KIR positivity in CRC tissue samples. One notable finding of our study was the variability in KIR staining intensity within the same tumor. We observed differences in KIR expression not only between tumors, but also within distinct areas of the same tumor. Additionally, a significant relationship was found between KIR expression and age.

**Conclusion:**

In conclusion, this study highlights the increased expression of both HLA-G and KIR markers in CRC patients, suggesting their potential as prognostic and predictive markers. Our findings also suggest that HLA-G and KIR molecules could represent valuable therapeutic targets for future cancer immunotherapy strategies.

## 1. Introduction

The immune system acts as a vital protective network, shielding the body from external threats as well as internal disruptions. In the context of cancer, tumor cells may express specific surface antigens that the immune system can recognize as foreign. This recognition has the potential to trigger an immune response. However, many cancer cells develop mechanisms to evade detection by reducing or entirely eliminating these surface antigens, making it challenging for the immune system to identify cancer cells as threats. For the immune system to function efficiently, the presence of both surface antigens and human leukocyte antigen (HLA) molecules is crucial, as they collaborate to help immune cells identify and attack cancerous cells.

A common strategy that tumor cells use to evade the immune system, particularly natural killer (NK) cells, involves modifications in HLA antigens [1–3]. These changes help the tumor cells avoid being identified and destroyed. Colorectal cancer (CRC), a prevalent and potentially fatal malignancy, disrupts various immune system processes [4, 5]. This includes altering HLA antigen expression on the cellular surface of tumors, impairing the function of NK cells, and facilitating immune evasion [6, 7]. These modifications can greatly impair the capability of the immune system to initiate a response against cancer cells [8, 9].

In the current study, the objective was to assess the soluble HLA-G (sHLA-G) in the blood serum of CRC patients using enzyme-linked immunosorbent assay (ELISA). Additionally, we examined the HLA-G expression loss in tumor tissues using immunohistochemistry (IHC), and we aimed to assess the infiltration of killer cell immunoglobulin-like receptors (KIRs) on NK cells within the tumor microenvironment. Through these analyses, our aim was to gain a deeper understanding of how CRC tumors avoid detection by the immune system and to identify potential therapeutic targets for future cancer therapies.

## 2. Materials and methods

### 2.1. Patient group

In this study, 36 individuals with a diagnosis of CRC were enrolled, including 24 males and 12 females. These patients underwent surgery at the General Surgery Clinic of İstanbul Bakırköy Dr Sadi Konuk Training and Research Hospital. The age range for the included patients was between 18 and 90 years. Blood samples were collected from each patient before surgery using serum-separating tubes. The collected samples were then preserved at −80 °C to be analyzed at a later stage. Tumor tissues were obtained during surgery and processed for IHC analysis.

The current study received approval from by the Clinical Research Ethics Committee of İstanbul University (approval date: 13 March 2017, protocol number: 2016/1350). Informed consent was obtained from all participants, and the research followed the ethical standards outlined in the Declaration of Helsinki.

### 2.2. Healthy control group

The control group consisted of 40 healthy volunteers, with 22 females and 18 males, all between the ages of 18 and 90 years. None of the individuals in this group had a history of acute or chronic illness, cancer, or previous treatments such as chemotherapy or radiotherapy. All healthy volunteers had undergone screening colonoscopies and provided informed consent for research purposes. Blood samples from the control participants were collected into tubes with EDTA, and the serum was preserved at −80 °C.

### 2.3. Immunohistochemistry

The colorectal tissue specimens were preserved using 10% neutral buffered formalin and were placed in paraffin blocks. Tissue slices were prepared from these blocks and transferred to glass slides. Samples were deparaffinized and rehydrated using a graded alcohol series. For IHC staining, the sections were exposed to primary antibodies targeting HLA-G (4H84, diluted 1:50, Santa Cruz Biotechnology, Dallas, TX, USA, catalog number: sc-21799) and KIR2DL4 Ab (rabbit polyclonal, diluted 1:7.5, Biorbyt, Cambridge, UK, catalog number: orb447529) according to the manufacturers’ guidelines. A negative control of PBS was applied instead of the primary antibody to check the immunolabelling, and all steps of the process were performed in the same manner. The slides were then treated using the hydrogen peroxide block/3-amino-9-ethyl-carbazole (HRP/AEC) antipolyvalent kit (Thermo Fisher Scientific, Waltham, MA, USA). A counterstain with hematoxylin was applied, and the stained slides were analyzed using a light microscope (Leica DM 2500, UK) coupled with a digital camera system (Leica DFC280, Germany). The staining intensity for each sample was categorized into 4 levels: no staining (−), weak staining (+), moderate staining (++), or strong staining (+++), following established criteria [10].

### 2.4. ELISA

Serum samples from CRC patients and healthy controls were analyzed for sHLA-G levels using ELISA. Prior to testing, the serum samples were centrifuged at 1000 rpm for 20 min. The Human HLA-G ELISA Kit (Abbkine Inc., China) was used following the manufacturer’s protocol [11], and absorbance was measured at 450 nm using an ELISA plate reader (Agilent, USA). The resulting data were used to determine sHLA-G concentrations in patient and control groups.

### 2.5. Statistical analysis

The data were processed using SPSS software version 21.0. For numerical variables, descriptive statistics (mean, standard deviation, minimum, and maximum values) were computed. Categorical variables were expressed as percentages. To check for normally distributed data, a Kolmogorov-Smirnov test was performed. For group comparisons, Student’s t-test was used when the data was normally distributed, whereas the Mann-Whitney U test was applied for nonnormally distributed data. Relationships between categorical variables were evaluated using Pearson’s chi-square test. A p-value below 0.05 was considered statistically significant.

## 3. Results

### 3.1. Demographic results

In this study, the CRC patient group was composed of 24 males (66.7%) and 12 females (33.3%), while the healthy control group included 18 males (45.0%) and 22 females (55.0%). The average age of the CRC patients was 62.33 ± 11.49 years, with an age range from 33 to 78. Conversely, the control group had a mean age of 50.03 ± 11.30, with ages ranging from 22 to 71.

### 3.2. HLA-G immunostaining results

IHC analysis was performed on tissue sections from 36 CRC patients, focusing on the expression of HLA-G. Cells displaying staining either on their membranes or within their cytoplasm were considered HLA-G positive. None of the 40 samples from the healthy control group were HLA-G positive. In the patient group, 16.7% (6 out of 36) of the tissue sections were HLA-G positive, while the remaining 83.3% (30 out of 36) were negative for HLA-G staining (Table 1). A statistically significant difference was observed between the CRC patients and healthy controls in terms of HLA-G expression (p = 0.009).

**Figure 1:**
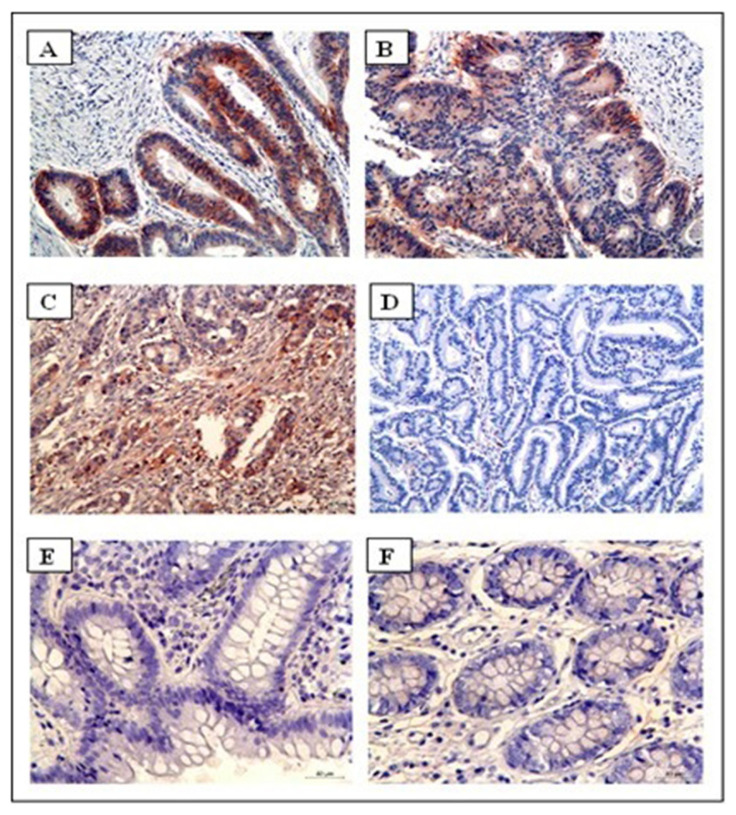
HLA-G immunostaining of patient and control samples. HLA-G positivity for CRC tissue with reddish staining (A, B, and C), HLA-G negativity for CRC tissue (D), and HLA-G negativity for control tissue (E and F).

The staining intensity of HLA-G in the CRC samples was categorized as weak (+), moderate (++), or strong (+++). Of the 6 positive cases, 1 (2.8% of all cancer patients) had weak staining (p = 0.473), 3 cases (8.3%) had moderate staining (p = 0.101), and 2 cases (5.6%) had strong staining (p = 0.221). The comparison of staining intensity between patient and control groups did not show any statistically significant differences. The IHC staining of HLA-G for patient and control groups is shown in Figure 1. Figures 1A, 1B, and 1C show tumor samples. Figures 1D shows HLA-G immunostaining negativity for healthy tissue. Figure 1C shows the strongest HLA-G positivity for tumor samples. Figure 1D shows HLA-G immunostaining negativity for tumour samples, while Figures 1E and 1F show HLA-G immunostaining negativity for healthy control tissue.

HLA-G expression was primarily observed in the cell membrane in 11.1% of the samples, while faint cytoplasmic staining was noted in 2.7% of the cases. These results indicate that while a subset of CRC patients were HLA-G positive, the majority did not show significant HLA-G expression in their tissue samples.

### 3.3. Correlation between HLA-G and clinicopathological features

We examined how HLA-G expression correlated with various clinical and pathological features, such as patient age, sex, tumor invasion depth, lymph node status, and tissue grade. No significant correlations were found between HLA-G positivity and these factors, including cancer stage (p = 0.658) (Table 2). When cancers were grouped into early and advanced stages, HLA-G expression was observed in 5.6% (2 out of 18) of early-stage patients and 11.1% (4 out of 18) of advanced-stage patients.

### 3.4. Killer cell immunoglobulin-like receptor (KIR) immunostaining results

**Figure 2:**
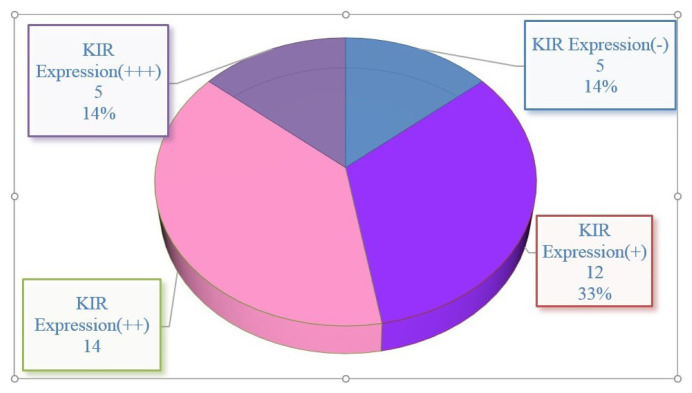
The distribution of KIR positivity expression in CRC patients. CRC patients were categorized as KIR (−), KIR (+), KIR (++), and KIR (+++). According to the IHC data, the sections of 5 patients (14%) were found to be KIR negative.

**Figure 3:**
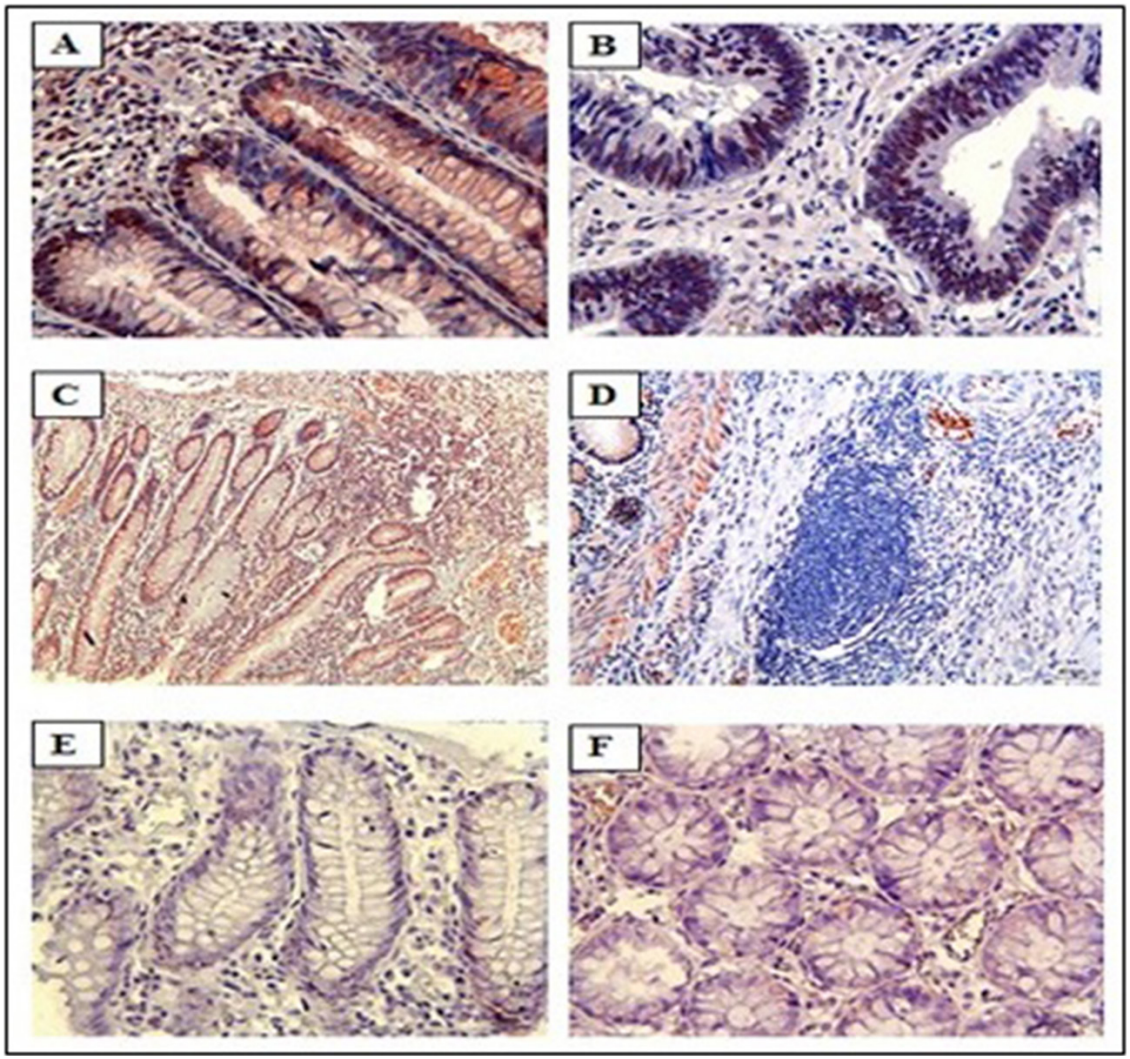
Positive and negative KIR staining in CRC and control tissues. KIR positivity for CRC tissue (A, B, and C), KIR negativity for CRC tissue (D), KIR negativity for control tissue (E and F).

**Figure 4:**
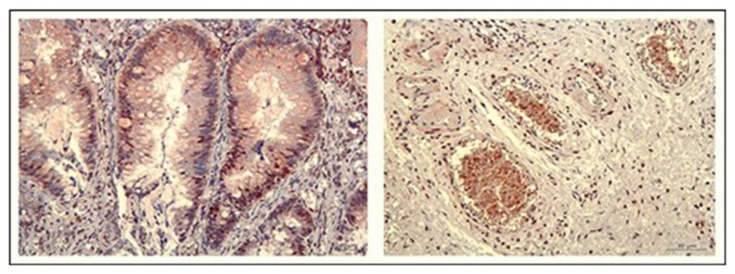
Heterogeneous KIR staining in CRC tissue. KIR expression varies from tumor to tumor and/or from one area to another within the same tumor. There are differences in the level of KIR expression between the tumors in the left and right images that were taken from different patients.

IHC staining for KIR expression had a significantly higher rate of KIR positivity in CRC tissue samples compared to control samples. Among the 36 CRC patient samples, 86% (31 out of 36) were positive for KIR staining. The staining intensity ranged from weak (+) to strong (+++), and heterogeneous staining patterns were observed across different regions of the same tumor. Figure 2 shows staining intensity. KIR expression was predominantly localized in the cell nucleus, with some cases also showing cytoplasmic staining. Representative microscopic images of IHC-stained samples are shown in Figure 3. Patients’ tumor samples can be seen in Figures 3A, 3B, and 3C. Figure 3D shows the negativity of KIR immunostaining for tumor samples. Figures 3E and 3F show the healthy control tissue. Figure 3A shows the strongest KIR positivity for tumor samples. A notable observation in our study was the variability in KIR staining intensity within the same tumor. Differences in KIR expression were observed not only between different tumors but also within distinct areas of the same tumor. KIR staining was mainly nuclear, although faint cytoplasmic staining was present in certain cases. Figure 4 shows KIR staining in tumor sites.

### 3.5. Correlation between KIR and clinicopathological features

We also investigated the relationship between KIR and clinicopathological factors, including tumor depth (T), lymph node (N), and metastasis (M) involvement as TNM stage. Among the 36 CRC patients, 83.3% of females and 87.5% of males were KIR positive, with no significant difference in KIR expression between sexes (p = 1). An analysis of the relationship between age (mean = 62.3 years) and KIR expression in the patient group showed that the mean age of individuals with KIR expression was 64.10 ± 10.36 years, whereas those without KIR expression had a mean age of 51.40 ± 0.35 years. This difference was statistically significant (p = 0.02). There were no meaningful associations identified between KIR and factors such as tumor stage, depth of invasion, or lymph node involvement.

### 3.6. Serum sHLA-G levels

Serum levels of sHLA-G were measured in both CRC patients and healthy controls. The median sHLA-G concentration in CRC patients was 227.78 ng/L, while the median concentration in the control group was 231.02 ng/L. No significant associations were observed between serum sHLA-G levels and clinicopathological factors, including cancer stage, tumor invasiveness, and the number of lymph nodes involved (p = 0.815).

### 3.7. Comparison of KIR, HLA-G, and sHLA-G

The analysis comparing KIR, HLA-G, and sHLA-G expression showed no notable correlation between KIR and sHLA-G levels (p = 0.707), nor between HLA-G and sHLA-G levels (p = 0.641). Among CRC patients, 86.1% were positive for KIR expression, whereas only 16.7% had HLA-G positivity. As no HLA-G or KIR expression was detected in the healthy control group, the significance of sHLA-G levels in controls was not analyzed further.

## 4. Discussion

CRC is recognized as a growing global health concern, with its incidence steadily increasing every year. Globally, CRC ranks as the third most diagnosed, while in Türkiye, it is the fourth most prevalent cancer [12, 13]. CRC is ranked second among cancer-related deaths globally [12, 14].

According to a 2023 report, more than 20 million new cancer cases were reported worldwide, with approximately 9.7 million deaths (48.5%), placing CRC as one of the leading causes of cancer mortality^1^.

Despite advancements in cancer treatment, the 5-year survival rate for CRC remains at approximately 65%^2^. These statistics emphasize the need for new therapeutic approaches, especially in the field of immunotherapy.

One of the key mechanisms that cancer cells use to evade immune system detection is the downregulation or alteration of surface antigens. These modifications enable tumor cells to avoid recognition and elimination by immune cells, including NK cells and cytotoxic T lymphocytes [15–17]. Effective immune surveillance depends on the proper expression of HLA on tumor cells. Alterations in HLA expression allow tumor cells to evade immune system responses, particularly those mediated by NK cells, which may lead to poor clinical outcomes [18–20].

Our study sought to investigate the expression of HLA-G and KIR markers in CRC tissues, as well as sHLA-G level in CRC patients. Our results showed a significant difference for HLA-G and KIR markers between CRC patients and healthy controls. Specifically, 16.7% of CRC tissue samples were HLA-G positive, while 86.1% were positive for KIR expression. These findings are consistent with previous research suggesting that increased HLA-G expression in tumor tissues is associated with immune evasion and a poor prognosis [21, 22]. The interaction between HLA-G and KIR likely plays a critical role in inhibiting NK cell activity, allowing tumor cells to evade destruction [23–25].

Several studies have reported elevated HLA-G expression in a variety of cancers, including lymphomas, lung cancer, and CRC [26–28]. For instance, a research group found that 65% of CRC tissue samples had HLA-G expression, while no expression was detected in normal colorectal tissues or benign adenomas [21]. Our findings are consistent with this, as we observed a higher expression of HLA-G in CRC tissues compared to healthy controls. The absence of HLA-G expression in healthy tissues further supports its role as a marker of malignant transformation and immune evasion [22].

In certain cancers, the absence of HLA-G expression is associated with better clinical outcomes, as it makes tumor cells more susceptible to lysis induced by NK cells [2]. The HLA-G positivity rate observed in our study (16.7%) was below that reported in previous studies. This could be attributed to the limited number of samples analyzed. Nevertheless, the strong association between HLA-G and KIR expression highlights their potential role in promoting immune evasion in CRC.

Interestingly, unlike some previous studies that have suggested elevated serum sHLA-G levels are correlated with poor prognosis and immune suppression [29, 30], we did not find a significant association between sHLA-G levels and clinicopathological factors in CRC patients. This discrepancy may be explained by differences in sample sizes, patient populations, or assay methodologies. In order to enhance our knowledge about the role of sHLA-G in CRC, future research involving larger patient groups are necessary.

In our analysis, a significant relationship between KIR expression and patient age was observed, with older patients showing higher levels of KIR positivity (p = 0.020). Despite this, no notable associations were identified between KIR and pathological features. The results indicate that age-related alterations in immune function could have an impact on KIR expression, though additional studies are needed to clarify the mechanisms involved.

Overall, our study highlights the importance of targeting HLA-G and KIR as potential therapeutic strategies for CRC. Blocking KIR receptors may enhance NK cell-mediated cytotoxicity, potentially improving clinical outcomes for CRC patients. Future research should focus on elucidating the mechanisms by which HLA-G and KIR interactions influence NK cell function and exploring how these pathways can be targeted for cancer immunotherapy.

## 5. Conclusion

The findings of this study indicate that HLA-G levels are markedly increased in CRC patients, indicating its critical role in helping tumor cells evade immune detection. By interacting with KIR receptors on NK cells, HLA-G contributes to the suppression of NK cell activity, allowing tumor cells to escape immune-mediated destruction. Although our lack of knowledge regarding the cell type responsible for KIR positivity in our findings is a significant limitation of our study, the use of methods such as double staining in future studies could resolve this uncertainty and support our belief that both HLA-G and KIR can serve as valuable prognostic markers.

Overall, our findings suggest that HLA-G and KIR have the potential to be promising targets for future cancer immunotherapies. Further research into their interactions with the immune system will be crucial for developing more effective treatments for CRC and improving patient outcomes. In particular, characterizing the genetic and functional properties of these interactions could contribute to the development of more targeted treatments against cancer.

## Figures and Tables

**Table 1:** HLA-G immunopositivity of patients.

	Number of samples	%	OR 95%	p-value
**Patient**			0.057 (0.003–1.069)	0.009
Staining +	6	16.7		
No staining	30	83.3		
Total	36	100		
**Control**				
Staining +	0	0		
No staining	40	100		
Total	40	100		

OR 95% = odds ratio

**Table 2:** Correlation of HLA-G expression and clinicopathologic parameters in the CRC group.

Clinic pathology	Number of samples	HLA-G expression		P-value
		Staining positive	Staining negative	
**Age**	36	66.33 ± 9.66	61.53 ± 11.8	0.358
**CRC patients**	36 (%100.0)	6 (%16.7)	30 (%83.3)	0.573
**Sex**				
**Female**	12 (%33.3)	3(%50.0)	9 (%30.0)	0.199
**Male**	24 (%66.7)	3(%50.0)	21 (%70.0)	
**Tumor invasiveness**				
**T1–2**	4 (%11.1)	1(%16.7)	3 (%10.0)	0.708
**T3**	24 (%66.7)	5(%83.3)	19 (%63.3)	
**T4**	8 (%22.2)	0 (%0.0)	8 (%26.7)	
**Number of lymph nodes**				
**N0**	18 (%50.0)	2(%33.3)	16 (%53.3)	0.409
**N1**	6(%16.7)	1(%16.7)	5 (%16.7)	
**N2**	12 (%33.3)	3(%50.0)	9 (%30.0)	
**TNM stage**				
**Early**	18(%50.0)	2(%33.3)	16(%53.3)	0.658
**Advanced**	18(%50.0)	4(%66.7)	14(%46.7)	

CRC Patients = colorectal cancer patients; TNM Stage = tumor depth (T), lymph node (N), and metastasis (M) involvement

## Footnotes

1 World Health Organization (2024). Global cancer burden growing, amidst mounting need for services [online]. Web site https://www.who.int/news/item/01-02-2024-global-cancer-burden-growing--amidst-mounting-need-for-services [accessed 20 September 2024].

2 American Cancer Society (2024). Key Statistics for Colorectal Cancer [online]. Web site https://www.cancer.net/cancer-types/colorectal-cancer/statistics [accessed 20 September 2024].
